# Biological observations in microbiota analysis are robust to the choice of 16S rRNA gene sequencing processing algorithm: case study on human milk microbiota

**DOI:** 10.1186/s12866-020-01949-7

**Published:** 2020-09-18

**Authors:** Shirin Moossavi, Faisal Atakora, Kelsey Fehr, Ehsan Khafipour

**Affiliations:** 1 Digestive Oncology Research Center, Digestive Disease Research Institute, Tehran University of Medical Sciences, Tehran, Iran; 2 Department of Medical Microbiology and Infectious Diseases, University of Manitoba, Winnipeg, MB Canada; 3 Children’s Hospital Research Institute of Manitoba, Winnipeg, MB Canada; 4 Developmental Origins of Chronic Diseases in Children Network (DEVOTION), Winnipeg, MB Canada; 5 Present Address Department of Physiology and Pharmacology & Mechanical and Manufacturing Engineering, University of Calgary, Calgary, AB Canada; 6 Department of Pediatrics and Child Health, University of Manitoba, Winnipeg, MB Canada; 7 Department of Animal Science, University of Manitoba, Winnipeg, MB Canada; 8 Present Address Microbiome Research and Technical Support, Cargill Animal Nutrition, Diamond V brand, Cedar Rapids, USA

**Keywords:** Qiime1, Qiime2, Decontam, Reproducibility, Microbiome, Milk microbiota, Human milk, CHILD cohort

## Abstract

**Background:**

In recent years, the microbiome field has undergone a shift from clustering-based methods of operational taxonomic unit (OTU) designation based on sequence similarity to denoising algorithms that identify exact amplicon sequence variants (ASVs), and methods to identify contaminating bacterial DNA sequences from low biomass samples have been developed. Although these methods improve accuracy when analyzing mock communities, their impact on real samples and downstream analysis of biological associations is less clear.

**Results:**

Here, we re-processed our recently published milk microbiota data using Qiime1 to identify OTUs, and Qiime2 to identify ASVs, with or without contaminant removal using *decontam.* Qiime2 resolved the mock community more accurately, primarily because Qiime1 failed to detect *Lactobacillus*. Qiime2 also considerably reduced the average number of ASVs detected in human milk samples (364 ± 145 OTUs vs. 170 ± 73 ASVs, *p* < 0.001). Compared to the richness, the estimated diversity measures had a similar range using both methods albeit statistically different (inverse Simpson index: 14.3 ± 8.5 vs. 15.6 ± 8.7, *p* = 0.031) and there was strong consistency and agreement for the relative abundances of the most abundant bacterial taxa, including *Staphylococcaceae* and *Streptococcaceae*. One notable exception was *Oxalobacteriaceae*, which was overrepresented using Qiime1 regardless of contaminant removal. Downstream statistical analyses were not impacted by the choice of algorithm in terms of the direction, strength, and significance of associations of host factors with bacterial diversity and overall community composition.

**Conclusion:**

Overall, the biological observations and conclusions were robust to the choice of the sequencing processing methods and contaminant removal.

## Background

Amplicon sequencing targeting bacterial 16S rRNA gene has so far been the most commonly used sequencing method for microbiome studies. In recent years there have been novel developments in several aspects of sequencing processing including a shift from clustering-based methods of operational taxonomic unit (OTU) designation based on sequence similarity (commonly > 97%) [1, 2] to denoising algorithms which identify exact amplicon sequence variants (ASVs) [3–9]; thereby increasing ecological precision. Performance of the denoising methods has been assessed mostly on mock communities [7–12]. However, the impact of these different methods on characterizing real biospecimens and downstream analysis of biological associations is less clear. The detection of true biological and ecological variations appears to be robust to the choice of sequencing processing method (OTU vs. ASV) in a few studies on soil, mouse feces, and human intestinal biopsies [12–14], but head-to-head comparisons are lacking for most human microbiota communities.

**Fig. 1:**
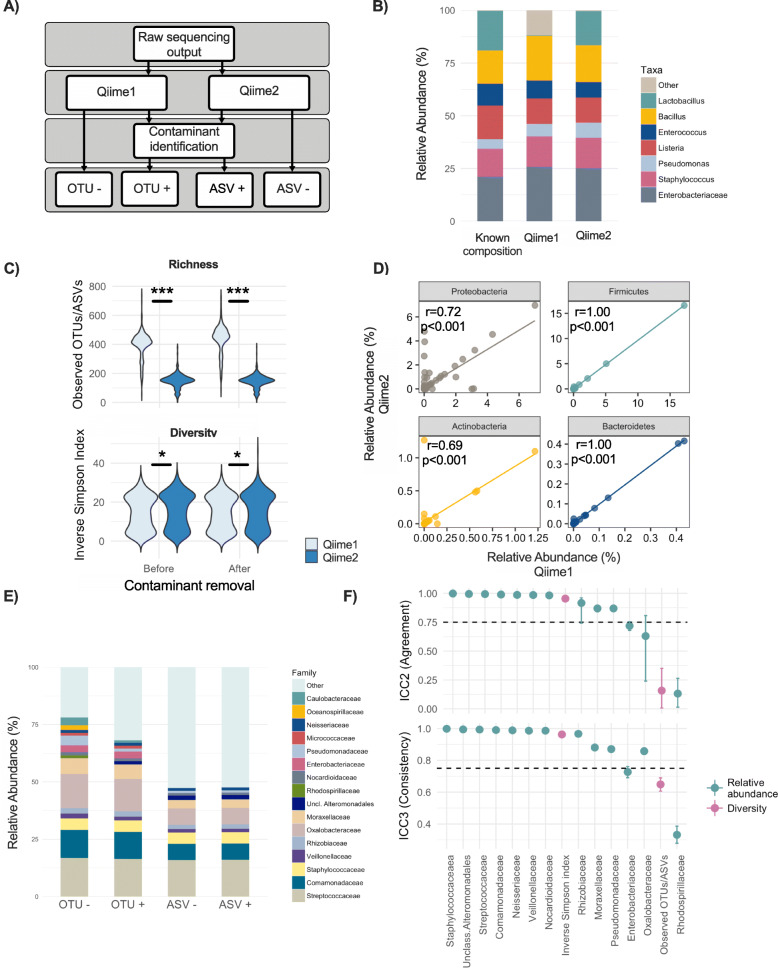
Microbiota composition in a mock community and human milk samples using a clustering-based method (Qiime1) and a denoising algorithm (Qiime2) with and without contaminant removal. **a** Schematic of the study design. **b** Composition of the mock community by Qiime1 and Qiime2 prior to contaminant removal (each dataset = combined data from 8 replicates). **c** Comparison of milk microbiota richness (observed OTUs/ASVs) and diversity (inverse Simpson index) between Qiime1 and Qiime2 with and without contaminant removal. **d** Correlation of the relative abundances of milk genera between Qiime1 and Qiime2 prior to contaminant removal (See also Figures. S2 and S3 and Tables S2 and S3) (each dataset = combined data from 393 milk samples). Each dot represents a classified genus. Contaminant removal doesn’t impact the associations (not shown). **e** Comparison of the composition of abundant families (> 1% mean relative abundance) between Qiime1 and Qiime2 with or without contaminant removal. Contaminant removal reduced the relative abundance of certain low-abundance taxa (e.g. *Caulobacteraceae* and *Rhodospirillaceae*) and proportion of Other taxa (OTUs with less than 1% mean relative abundance) estimated by Qiime1, but generally did not affect the microbiota profile estimated by Qiime2. **f** Agreement and consistency between methods by intraclass correlation for alpha diversity and 13 most abundant families. * *p* < 0.05, *** *p* < 0.001

Another issue receiving increasing attention in sequencing-based microbiome analysis is contamination introduced during DNA extraction and library preparation. This is especially of concern for low biomass samples where the signal-to-noise ratio is very low [15, 16]. In this case, reproducible downstream analyses plausibly depend on the identification and removal of potential contaminants. Milk is a low biomass sample and thus highly susceptible to reagent contaminants in culture-independent sequencing-based microbiota profiling [17]. To our knowledge, the majority of previously published milk microbiota studies are based on OTU-picking methods and have not assessed the potential reagent contaminants [18]. Therefore, the comparability and generalizability of different studies in terms of the milk microbiota composition and association with maternal and infant characteristics are not known. To address these knowledge gaps, we re-processed our recently published 16S rRNA gene sequencing milk microbiota dataset [19] using Qiime1 closed-reference OTU picking and Qiime2 denoising method with or without contaminant removal using *decontam* [20]. We adhered to the quality control process and taxonomy assignment threshold commonly used by these methods (97% for Qiime1 and 99% for Qiime2) to examine the real world impact of these different approaches on downstream analysis. The datasets resulting from these four approaches (Fig. 1a) were used to assess the comparability of results in terms of microbiota features (taxonomy, alpha, and beta diversity) and test the hypothesis that biological associations are robust to the choice of upstream data processing.

## Results

We analyzed 18 replicates (3 per PCR plate in 2 sequencing runs) of a mock community consisting of 8 different bacterial species with a known composition (ZymoBIOMICS™ Microbial Community Standard, Zymo Research, USA). While the Qiime1 method detected an average (SD) of 223 (50) OTUs in the mock community samples, Qiime2 performance was closer to the expected composition, detecting 12 (3) ASVs (Table S1). Although contaminant removal did not considerably reduce the number of OTUs, it decreased the average (SD) ASVs to 9 (3) effectively eliminating the potential contaminants (Table S1). Overall, there was a good agreement between the observed and expected taxonomic composition with both methods (Fig. 1b). However, two notable differences were observed. Most prominently, Qiime2 performed better at identifying *Lactobacillus:* the actual contribution of this genus to the mock community was 19%; the estimated relative abundance was < 1% using Qiime1 compared to 16% using Qiime2. Moreover, the proportion of identified taxa not belonging to the mock community (likely contaminants) was higher with Qiime1 (~ 12% vs. 0.1% in Qiime2). Neither method could accurately identify *Escherichia coli* or *Salmonella enterica* present in the mock community; however, for both methods, the relative abundance of taxa identified as *Enterobacteriaceae* was within the range of the combined relative abundances of these two enteric bacteria. Thus, overall, Qiime2 provided a more accurate representation of the mock community (Table S1 and Fig. 1b) in agreement with previous studies [7–12].

Overall when comparing the four approaches, the mean depth of sequencing per sample was slightly lower in Qiime2 compared to Qiime1 both before and after contaminant removal. The differences in library size within each method before and after *decontam* were negligible (Figure S1). Despite differences in the initial number of OTUs/ASVs in total and on average, Qiime1 and Qiime2 resulted in a similar number of remaining OTUs/ASVs (298 and 299 respectively before contaminant removal) after filtering taxa with less than 0.01% mean relative abundance (Table S1). This suggests the majority of “noisy true reads” (true reads containing sequencing errors [10]) initially retained by Qiime1 were eliminated by applying abundance-based filtering. In agreement with the literature [10, 12], there was a considerable difference in the number of observed OTUs/ASVs prior to filtering very low abundant taxa (364 ± 145 average OTUs vs. 170 ± 73 average ASVs per sample, Table S1). The bacterial richness at OTU/ASV level remained higher in Qiime1 vs. Qiime2 even after data filtering, regardless of contaminant removal (394 ± 91 vs. 148 ± 44, *p* < 0.001) (Fig. 1c). In contrast, milk microbiota diversity was slightly but significantly higher with Qiime2 regardless of contaminant removal (14.3 ± 8.5 vs. 15.6 ± 8.7 *p* < 0.05) (Fig. 1c) suggesting that the number of the abundant taxa remained consistent in both Qiime1 and Qiime2 methods.

Next, we compared the relative abundance (Fig. 1d) and prevalence (Figure S2) of genera belonging to the major milk phyla (Figure S3 and Table S2) between methods and observed high degrees of correlation, especially for Firmicutes, Actinobacteria, and Bacteroidetes (Table S3). In comparing abundant genera (> 0.01% mean relative abundance) (Figure S3 and Table S4), relative abundances remained highly correlated (Figure S4). Overall, the relative abundances of the most abundant families (> 1% mean relative abundance) including *Streptococcaceae* and *Staphylococcaceae* were not considerably impacted by the choice of the sequencing processing method, with and without contaminant removal (Fig. 1e and Figure S5). However, there were notable differences between methods for some other taxa. For example, at the family level, *Oxalobacteriaceae* was detected at higher relative abundances by Qiime1 compared to Qiime2 (14.1% ± 8.4% vs. 7.2% ± 4.1%, Figure S5), while *Enterobacteriaceae* and *Caulobacteraceae* were only observed as top abundant families using Qiime1 (Fig. 1e). In addition, *Oxalobacteriaceae* and *Comamonadaceae* were not assigned taxonomy at genus level using Qiime1, whereas some members of these families including *Acidovorax* (family *Comamonadaceae*), *Ralstonia*, and *Massilia* (*Oxalobacteriaceae*) were resolved by Qiime2. In contrast, *Methylibium* (family *Comamonadaceae*) and *Erwinia* (family *Enterobacteriaceae*) were only identified by Qiime1 as abundant taxa. Overall, the total proportion of less abundant OTUs/ASVs (< 1% mean relative abundance) was higher in Qiime2 (Fig. 1e) while the number of true abundant taxa was less biased, possibly due to the lack of binning of multiple similar sequence variants into an OTU. In agreement with this interpretation, contaminant removal considerably increased the proportion of less abundant taxa only in Qiime1 suggesting that some of the abundant taxa were consistent of contaminants (Table S1).

Overall, agreement and consistency between different methods (Fig. 1f) were quite low for milk microbiota richness, highlighting the sensitivity of this measure to the choice of bioinformatics method. Nevertheless, a very high inter-class correlation for inverse Simpson index (0.95) and relative abundances for the majority of the abundant families (above 0.75 for 10/13 families) suggests an acceptable degree of agreement and consistency, which would be required for downstream analyses to generate comparable results.

**Fig. 2:**
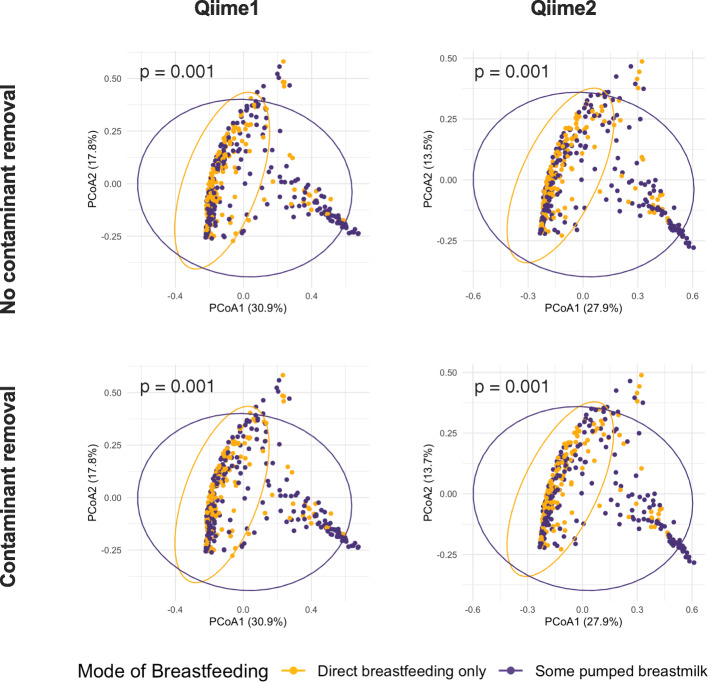
Impact of four sequence processing approaches on the association of mode of breastfeeding with milk microbiota beta diversity. We re-processed our published 16S rRNA gene sequencing milk microbiota dataset [19] using Qiime1 and Qiime2 with or without contaminant removal resulting in four datasets (see also Fig. 1a). The Association of mode of breastfeeding with milk microbiota beta diversity was assessed on Bray-Curtis dissimilarity matrix and was tested by permutational ANOVA (PERMANOVA)

**Fig. 3:**
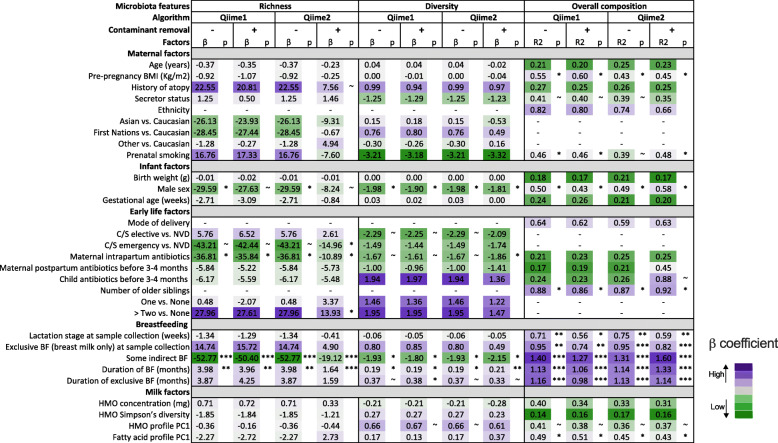
Impact of four sequence processing approaches on observed associations of milk microbiota richness (observed OTUs/ASVs), diversity (inverse Simpson index), and overall composition with maternal, infant, early life, breastfeeding, and milk factors. We re-processed our published 16S rRNA gene sequencing milk microbiota dataset [19] using Qiime1 and Qiime2 with or without contaminant removal resulting in four datasets (see also Fig. 1a). Beta coefficients from univariate linear regression (richness and diversity) or R^2^ from redundancy analysis (overall composition) are presented and colour coded within each microbiota feature. Results of Qiime2 with contaminant removal are originally reported in Moossavi et al. [19]. BF, breastfeeding; BMI, body mass index; C/S, Cesarean section; HMO, human milk oligosaccharide; NVD, normal vaginal delivery; PC1, Principal Component 1 * *p* < 0.05, ** *p* < 0.01, *** *p* < 0.001

Given the differences in microbiota alpha diversity and taxonomic structure resulting from the choice of processing approaches (Fig. 1), we explored whether the processing approach also influenced the association of microbiota and metadata variables. To do this, we 1) assessed the association of mode of breastfeeding with milk microbiota beta diversity and 2) compared the association of maternal, infant, and early life factors, breastfeeding practices, and other milk components with milk microbiota richness, diversity, and overall composition as previously described [19]. We have previously identified mode of breastfeeding to be significantly associated with milk microbiota beta diversity [19]. Here, we observed similar beta diversity association patterns with mode of breastfeeding regardless of the sequencing processing method or contaminant removal (Fig. 2). Overall, there were high degrees of concordance in the direction, strength, and significance of association between milk microbiota diversity and overall composition with the independent variables assessed using both methods, regardless of contaminant removal (Fig. 3). However, some method-related differences were observed for associations with microbiota richness. While the direction and strength of associations using Qiime2 without contaminant removal were comparable to the Qiime1, contaminant removal generally resulted in weaker associations and lower effect size estimates when using Qiime2-processed data (e.g. for maternal atopy, infant sex, and mode of breastfeeding). Occasionally, the direction of association was also different in Qiime2/decontaminated compared to the other processing methods (e.g. for prenatal smoking and fatty acids profile) (Fig. 3).

## Discussion

We compared the milk microbiota composition and associations with maternal, infant, early life, and milk factors on data processed by a clustering-based method into OTUs vs. a denoising method resulting in ASVs. Additionally, we compared the impact of contaminant removal on the statistical conclusions. Overall, richness was strongly impacted by the choice of the bioinformatics approach while statistical contaminant removal had minimal additional impact. There was an acceptable agreement and consistency in the relative abundances of the dominant milk bacteria and milk diversity. Additionally, the main conclusions remained robust to the choice of data processing.

Sequencing-based microbiome studies are highly influenced by the various bioinformatics and data processing choices [21, 22]; specifically, with the recent shift from clustering-based OTU picking methods to denoising algorithms identifying ASVs with higher ecological accuracy. Generally, it is not clear how the results of previously published milk microbiota studies using OTU-picking methods would compare to the more recent results including ours using ASV methods. In a head-to-head comparison of an OTU-picking method with a denoising algorithm, we observed high degrees of agreement and consistency in milk microbial features regardless of the methods. Our results suggest that although milk microbiota richness and some members of the microbial community are strongly influenced by the choice of sequencing processing method, there is high agreement and consistency between methods for estimating microbiota diversity and quantifying the majority of the most abundant taxa. Addition of a contaminant removal step resulted in minor shifts in the composition of the most abundant taxa as well as association of richness with a few of the variables assessed when using Qiime2-processed data. The overall conclusions of the study and the main determinants of milk microbiota composition (e.g. consistent association of feeding mode with milk microbiota composition [19]) remained robust to the choice of OTU vs. ASV methods with or without contaminant removal.

While the overall agreement between different data processing approaches was high, some differences stood out. For example, the relative abundance of *Oxalobacteriaceae*, an environmental bacteria and a common reagent contaminant, was lower using denoising methods, potentially in line with higher classification accuracy of the denoising approaches. Notably, there was not a difference in the relative abundance of *Oxalobacteriaceae* before and after identification and removal of potential reagent contaminants using *decontam*, potentially highlighting the limitation of these methods for low biomass samples [23]. Computational methods, while helpful, do not replace the need for careful study design, sample handling, and reagent controls. Additionally, culture-based methods such as culture-enriched molecular profiling may inform the sequencing results of low biomass samples [24].

While new bioinformatic methods are typically evaluated using mock communities, we have provided a real-word comparison of two commonly used sequencing processing approaches (Qiime1 vs. Qiime2). A limitation of our study is that both approaches are sequencing-dependent and prone to reagent contaminants, and therefore neither can verifiably identify “true” milk taxa. Thus, while we have provided evidence that the statistical results obtained by the two approaches are comparable, it is important that the microbiological and ecological implications be studied using controlled experimental designs.

## Conclusion

In summary, we have shown that Qiime2 resolved the mock community more accurately and there were high degrees of agreement and consistency in milk microbiota features regardless of the choice of the sequencing processing approach (OTU vs. ASV). In light of our observation that the associations with metadata and the main conclusions were robust to the choice of sequence processing approaches and contamination removal, previous studies of milk microbiota and potentially other low biomass samples using OTU picking approaches are likely valid both in terms of the composition of the abundant taxa and associations, especially for metrics that put less emphasis on richness.

## Methods

### Study design

We used our published data on milk microbiota [19] (SRA accession number: PRJNA481046) in the CHILD cohort [25]. Each mother provided one sample of milk at 3–4 months postpartum [mean (SD) 17 (5) weeks postpartum] in a sterile milk container provided by the CHILD study. Milk microbiota was profiled by sequencing the V4 hypervariable region of 16S rRNA gene on a MiSeq platform (Illumina, San Diego, CA, USA) as previously described [19].

### Microbiome sequencing processing

Overlapping paired-end reads were separately processed with a clustering-based (Qiime1) and a denoising algorithm (Qiime2). In the clustering-based approach, paired-end reads were merged using the PANDAseq assembler [26]. Sequences with low quality base calling scores (< 20) as well as those containing ambiguous bases in the overlapping region were discarded. The subsequent fastq file was processed using the open-source software Qiime v1.9.1 [27]. Assembled reads were demultiplexed according to the barcode sequences and chimeric reads were filtered using UCHIME [28]. Reads were clustered into OTUs using closed-reference OTU picking based on 97% similarity using UCLUST [29]. Representative sequences from each OTU were assigned a taxonomy using RDP Classifier [30] and aligned to the 2013 release of the Greengenes reference database at 97% sequence similarity [31] using PyNAST [32]. In the denoising approach, overlapping paired-end reads were processed with DADA2 pipeline [7] using the open-source software Qiime 2 v.2018.6 (https://Qiime2.org) [27]. Unique ASVs were assigned taxonomy and aligned to the 2013 release of the Greengenes reference database at 99% sequence similarity [31].

### OTU/ASV table pre-processing and filtering

Initial pre-processing of the OTU/ASV table was conducted using the Phyloseq package [33]. As previously reported [19], the mean (SD) sequencing depth was 47,710 (18,643). Samples with less than 25,000 sequencing reads were excluded (*n* = 35) and the remaining samples (*n* = 393) were rarefied to the minimum 25,000 sequencing reads per sample. OTUs/ASVs only present in the mock community or negative controls and OTUs/ASVs belonging to phylum Cyanobacteria, family of mitochondria, and class of chloroplast were removed. OTUs/ASVs with less than 20 reads across the entire dataset (*n* = 393 samples) were also removed. The numbers of sequencing reads of taxa were then relativized to the total sum of 25,000. This dataset was used for analysis unless otherwise specified.

### Reagent contaminant removal

Potential reagent contaminants were identified using *decontam* package based on either the frequency of the OTUs/ASVs in the negative control or the negative correlation with DNA concentration using default parameters [20].

### Performance assessment on mock community

The baseline performance of each method was assessed on DNA extracted from a high biomass mock community consisting of 8 bacterial species with known relative abundances (ZymoBIOMICS™ Microbial Community Standard, Zymo Research, USA).

### Statistical analysis

Depth of sequencing and alpha diversity (observed OTUs/ASVs and inverse Simpson index) were compared between methods (*n* = 4 datasets) using Student’s *t* test. Within the 4 most abundant phyla, the prevalence (percentage of samples containing the taxa) and average relative abundance of classified genera were compared between Qiime1 and Qiime2 prior to contaminant removal using Pearson correlation. Agreement and consistency of community alpha diversity and relative abundances of the most abundant families were assessed by interclass correlation by 2-way random and fixed single measurement models using Psych package [34, 35]. Association of mode of breastfeeding with milk microbiota beta diversity was assessed on Bray-Curtis dissimilarity matrix and was tested by permutational ANOVA (PERMANOVA) using the vegan package [36]. Separately for each method (within the *n* = 393 milk samples), the association of maternal, infant, early life, breastfeeding, and milk factors was assessed by linear regression (for microbiota alpha diversity) and redundancy analysis (RDA, for microbiota composition). RDA was performed with 1000 permutations using the vegan package [36] following zero-replacement and centre log-ratio transformation [37, 38].

## Supplementary information

**Additional file 1: Table S1.** Comparison of the number of OTUs/ASVs in the mock community, negative controls, and milk microbiota datasets processed by clustering-based OTU method (Qiime1) and a denoising algorithm (Qiime2) with or without contaminant removal. **Table S3.** Comparison of prevalence and relative abundance of shared genera in milk microbiota processed by clustering-based OTU method (Qiime1) and a denoising algorithm (Qiime2) without contaminant removal. **Figure S1.** Comparison of the library size on datasets processed by clustering-based OTU method (Qiime1) and a denoising algorithm (Qiime2) with or without contaminant removal. **Figure S2.** Comparison of the prevalence of bacterial genera on datasets processed by clustering-based OTU method (Qiime1) and a denoising algorithm (Qiime2) without contaminant removal. **Figure S3.** Distribution of classified genera in Qiime1 and Qiime2 processed datasets without contaminant removal. **Figure S4.** Comparison of the relative abundance and prevalence of abundant bacterial genera (> 0.01% mean relative abundance) on datasets processed by clustering-based OTU method (Qiime1) and a denoising algorithm (Qiime2) without contaminant removal. **Figure S5.** Comparison of the composition of abundant taxa (> 1% mean relative abundance) on datasets processed by clustering-based OTU method (Qiime1) and a denoising algorithm (Qiime2) with or without contaminant removal.

**Additional file 2: Table S2.** Prevalence of classified genera in datasets processed by Qiime1 and Qiime2 after excluding OTUs/ASVs with less than 20 reads across each dataset. **Table S4.** Prevalence of classified genera in datasets processed by Qiime1 and Qiime2 after excluding OTUs/ASVs with less than 20 reads across each dataset and mean relative abundance of less than 0.01%.

## Data Availability

The datasets generated and analysed during the current study are available in the Sequence Read Archive of NCBI repository (accession number PRJNA481046).

## Abbreviations

ASV: Amplicon sequence variants
OTU: Operational taxonomic unit
PCR: Polymerase chain reaction
RDA: Redundancy analysis
SD: Standard deviation
