# Supplementary material for: Biological observations in microbiota analysis are robust to the choice of 16S rRNA gene sequencing processing algorithm: case study on human milk microbiota
Source: BMC Microbiol. 2020 Sep 18;20:290. doi: 10.1186/s12866-020-01949-7 (PMC7501722; doi:10.1186/s12866-020-01949-7)

Supplementary Material for "Biological observations in microbiota analysis are robust to the choice of 16S rRNA gene sequencing processing algorithm: case study on human milk microbiota" (Moossavi et. al.)

Supplementary Tables & Figures

Table S1. Comparison of the number of OTUs/ASVs in the mock community, negative controls, and milk microbiota datasets processed by clustering-based OTU method (Qiime1) and a denoising algorithm (Qiime2) with or without contaminant removal.

Table S2. Prevalence of classified genera in datasets processed by Qiime1 and Qiime2 after excluding OTUs/ASVs with less than 20 reads across each dataset (See the supplementary excel file).

Table S3. Comparison of prevalence and relative abundance of shared genera in milk microbiota processed by clustering-based OTU method (Qiime1) and a denoising algorithm (Qiime2) without contaminant removal.

Table S4. Prevalence of classified genera in datasets processed by Qiime1 and Qiime2 after excluding OTUs/ASVs with less than 20 reads across each dataset and mean relative abundance of less than 0.01% (See the supplementary excel file).

Figure S1. Comparison of the library size on datasets processed by clustering-based OTU method (Qiime1) and a denoising algorithm (Qiime2) with or without contaminant removal.

Figure S2. Comparison of the prevalence of bacterial genera on datasets processed by clustering-based OTU method (Qiime1) and a denoising algorithm (Qiime2) without contaminant removal.

Figure S3. Distribution of classified genera in Qiime1 and Qiime2 processed datasets without

contaminant removal.

Figure S4. Comparison of the relative abundance and prevalence of abundant bacterial genera (>0.01% mean relative abundance) on datasets processed by clustering-based OTU method (Qiime1) and a denoising algorithm (Qiime2) without contaminant removal.

Figure S5. Comparison of the composition of abundant taxa (>1% mean relative abundance) on datasets processed by clustering-based OTU method (Qiime1) and a denoising algorithm (Qiime2) with or without contaminant removal.

Table S1. Comparison of the number of OTUs/ASVs in the mock community, negative controls, and milk microbiota datasets processed by clustering-based OTU method (Qiime1) and a denoising algorithm (Qiime2) with or without contaminant removal

	**Qiime1**		**Qiime2**	
Sequence filtering [a]	Yes		No	
Reference database	Greengenes		Greengenes	
Similarity threshold for taxonomy assignment	97%		99%	
Contaminant identification & removal	-	+	-	+
Sequencing depth (x1000)	51.1±21.2	51.0±21.1	48.6±19.2	47.7±18.6
Mock community of 8 species (N=18 [b])				
Average Number of OTUs/ASVs per sample	223±50	215±49	12±3	9±3
Negative control (N=15)				
Average Number of OTUs/ASVs per sample	58±94	14±20	42±65	25±35
Milk samples (N=428)				
Total number of OTUs/ASVs	13,305	12,695	9,884	9,711
Average Number of OTUs/ASVs per sample	364±145	358±132	170±73	168±70
Milk samples after filtering (N=393) [c]				
Remaining OTUs/ASVs after filtering	2,265	2,134	1,956	1,972
Average Number of OTUs/ASVs per sample	394±91	445±142	149±44	147±44
OTUs/ASVs > 0.01% average relative abundance [d]	298	298	299	301
Average number of OTUs/ASVs per sample after additional filtering [de]	18±7	18±7	18±6	18±6

[a] Low quality base calling scores (<20) and containing ambiguous bases in the overlapping region

[b] 3 replicates per PCR reaction (n=3) for each sequencing run (n=2)

[c] Exclusion of OTUs/ASVs not present in the samples or belonging to non-bacterial taxa, rarefaction to 25000 reads per sample, filtering OTUs/ASVs with less than 20 reads across each dataset

[d] Despite the similarity in the number of OTUs/ASVs, the composition is not identical (See **Figure 1**, **S4**, and **S5**).

[e] Excluding OTUs/ASVs with less than <1% mean relative abundance

Table S3. Comparison of prevalence and relative abundance of shared genera in milk microbiota processed by clustering-based OTU method (Qiime1) and a denoising algorithm (Qiime2) without contaminant removal.

Features	Method	Phylum			
		**Proteobacteria**	**Firmicutes**	**Actinobacteria**	**Bacteroides**
**OTUs/ASVs with more than 20 total read count across each dataset**					
OTUs/ASVs	**Qiime1**	N=2265			
	**Qiime2**	N =1956			
OTUs/ASVs with genus classification	**Qiime1**	N=1385			
	**Qiime2**	N=1378			
Genera common to both / total	N_{total} = 219/343	100/163	55/83	40/61	24/36
Unique genera / total for methods	**Qiime1**	26/126	11/66	26/66	6/30
	**Qiime2**	37/137	17/72	8/48	6/30
Correlation between methods (95% CI) *	Prevalenc e	r=0.66 (0.56, 0.74) p<0.001	r=0.79 (0.70, 0.86) p<0.001	r=0.58 (0.38, 0.73) p<0.001	r=0.80 (0.64, 0.89) p<0.001
	Mean Relative abundance	r=0.72 (0.63, 0.78) p<0.001	r=1 (1, 1) p<0.001	r=0.69 (0.53, 0.80) p<0.001	r=1 (1,1) p<0.001
**OTUs/ASVs with at least 0.01% mean relative abundance**					
OTUs/ASVs	**Qiime1**	N=298			
	**Qiime2**	N =299			
OTUs/ASVs with genus classification	**Qiime1**	N=195			
	**Qiime2**	N=234			
Genera common to both / total	N_{total} = 65/106	40/67	11/17	8/12	6/10
Unique Genera / total for algorithm	**Qiime1**	9/49	3/14	1/9	3/9
	**Qiime2**	18/58	3/14	3/11	1/7
Correlation between methods (95% CI) *	Prevalenc e	r=0.30 (0.06, 0.50) p=0.015	r=0.61 (0.19, 0.84) p=0.009	r=0.07 (–0.52, 0.62) p=0.8	r=0.66 (0.05, 0.91) p=0.037
	Mean Relative abundance	r=0.68 (0.52, 0.79) p<0.001	r=1 (1,1) p<0.001	r=0.55 (–0.03, 0.86) P=0.061	r=1 (0.99, 1) p<0.001

*Correlation was assessed using Pearson correlation using the shared genera present in both methods (See also **Figure 1C**, **S2-S4**, and **Table S2** and **S4**).

Figure S1. Comparison of the library size on datasets processed by clustering-based OTU method (Qiime1) and a denoising algorithm (Qiime2) with or without contaminant removal. ~ p<0.1, * p<0.05

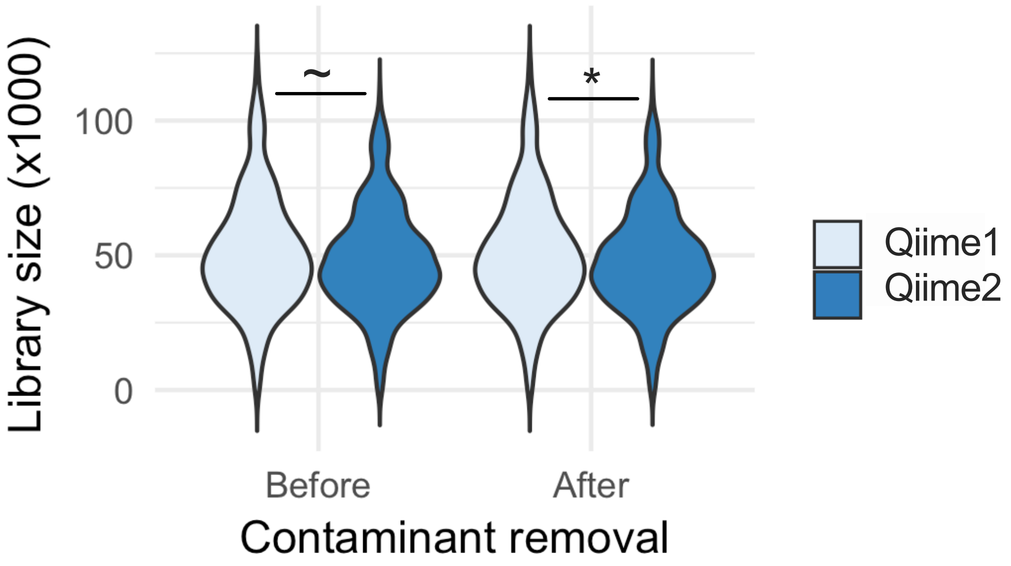

Figure S2. Comparison of the prevalence of bacterial genera on datasets processed by clustering-based OTU method (Qiime1) and a denoising algorithm (Qiime2) without contaminant removal. OTUs/ASVs with less than 20 total read count across each dataset and unclassified genera were excluded (n=234 shared genera included in the analysis). Each dot represents a classified genus. Contaminant removal doesn't impact the associations (not shown).

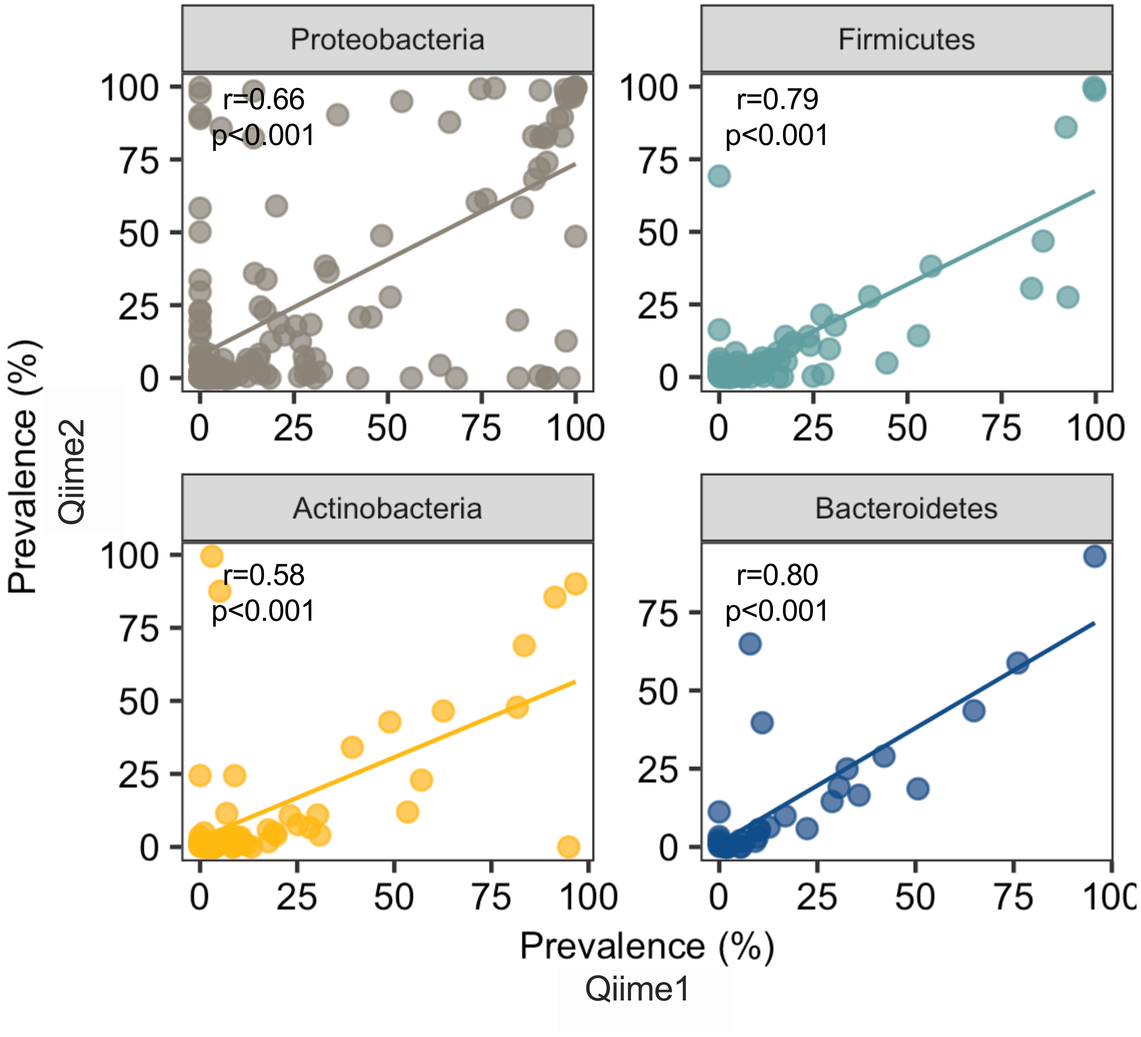

Figure S3. Distribution of classified genera in Qiime1 and Qiime2 processed datasets without contaminant removal. A) OTUs/ASVs > 20 total read count across each dataset. B) OTUs/ASVs > 0.01% mean relative abundance across each dataset.

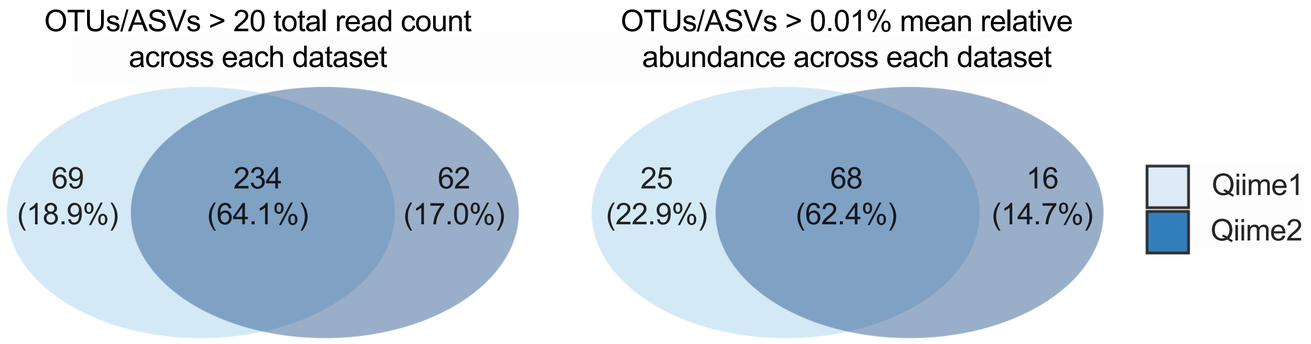

Figure S4. Comparison of the relative abundance and prevalence of abundant bacterial genera (>0.01% mean relative abundance) on datasets processed by clustering-based OTU method (Qiime1) and a denoising algorithm (Qiime2) without contaminant removal. Unclassified genera were excluded (n=68 shared genera included in the analysis).

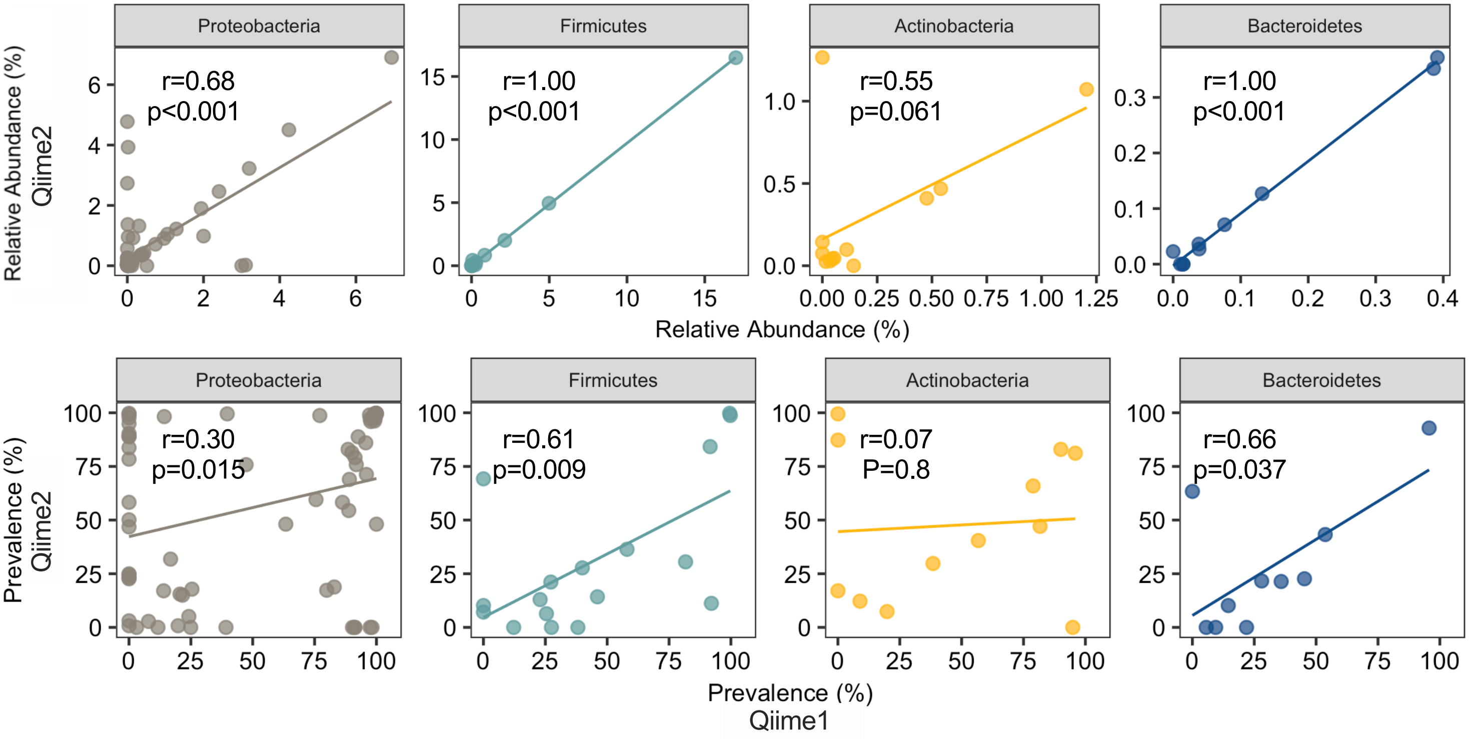

Figure S5. Comparison of the relative abundances of four abundant families (>1% mean relative abundance) on datasets processed by clustering-based OTU method (Qiime1) and a denoising algorithm (Qiime2) with or without contaminant removal. ANOVA test. *** $p<0.001$

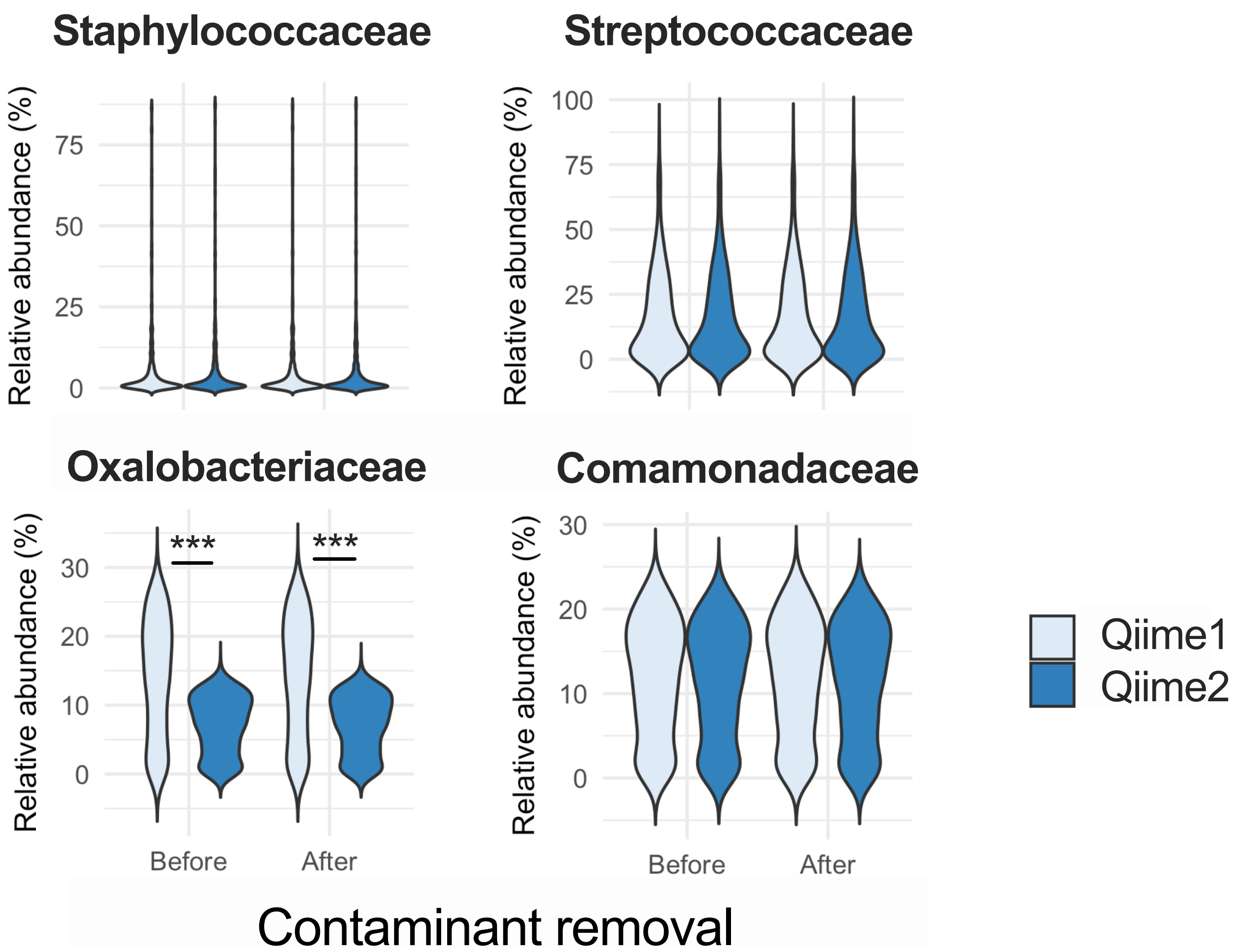

Supplement: Supplementary file 1 — Additional file 1: Table S1. Comparison of the number of OTUs/ASVs in the mock community, negative controls, and milk microbiota datasets processed by clustering-based OTU method (Qiime1) and a denoising algorithm (Qiime2) with or without contaminant removal. Table S3. Comparison of prevalence and relative abundance of shared genera in milk microbiota processed by clustering-based OTU method (Qiime1) and a denoising algorithm (Qiime2) without contaminant removal. Figure S1. Comparison of the library size on datasets processed by clustering-based OTU method (Qiime1) and a denoising algorithm (Qiime2) with or without contaminant removal. Figure S2. Comparison of the prevalence of bacterial genera on datasets processed by clustering-based OTU method (Qiime1) and a denoising algorithm (Qiime2) without contaminant removal. Figure S3. Distribution of classified genera in Qiime1 and Qiime2 processed datasets without contaminant removal. Figure S4. Comparison of the relative abundance and prevalence of abundant bacterial genera (> 0.01% mean relative abundance) on datasets processed by clustering-based OTU method (Qiime1) and a denoising algorithm (Qiime2) without contaminant removal. Figure S5. Comparison of the composition of abundant taxa (> 1% mean relative abundance) on datasets processed by clustering-based OTU method (Qiime1) and a denoising algorithm (Qiime2) with or without contaminant removal. [file 12866_2020_1949_MOESM1_ESM.pdf]
